# Anti-Inflammatory Effects of the Fraction from the Leaves of *Pyrus pyrifolia* on LPS-Stimulated THP-1 Cells

**DOI:** 10.1155/2021/4946241

**Published:** 2021-08-25

**Authors:** Gilhye Lee, Jung-Hee Kim, Hyun-Jae Jang, Ji-Won Park, Jae-Won Lee, Ok-Kyoung Kwon, Jae-Hong Kim, Kyung-Seop Ahn

**Affiliations:** ^1^Natural Medicine Research Center, Korea Research Institute of Bioscience and Biotechnology, Cheongju-si 28116, Republic of Korea; ^2^College of Life Science and Biotechnology, Korea University, Seoul 02841, Republic of Korea

## Abstract

*Pyrus pyrifolia* Nakai (*P. pyrifolia*) has been traditionally used in East Asia to treat diseases such as phlegm, cough, hangover, and fever. However, there is no investigation that evaluates the biological activities of the leaves of *P. pyrifolia.* This study aims at describing the anti-inflammatory effects of PP, a bioactive fraction from the leaves of *P. pyrifolia*, in lipopolysaccharide (LPS)-stimulated THP-1 cells. Initially, PP decreased the protein and RNA expression of TNF-*α*, MCP-1, IL-8, and IL-6 induced by LPS. Moreover, PP attenuated the phosphorylation of p38, JNK, and ERK. In addition, after stimulation with LPS, the degradation of I*κ*B-*α* was suppressed by PP, and the phosphorylation of I*κ*B-*α* and p65 was suppressed by PP. Additionally, PP increased HO-1, which controls the production of inflammatory molecules, by activating Nrf2. These results indicated that PP could be used as an anti-inflammatory drug to promote wellness.

## 1. Introduction

Inflammation is one of the biological defences against harmful stimuli and risk factors such as pathogens [1] and is involved in numerous human diseases like restenosis, hypertension, and atherosclerosis [2]. Lipopolysaccharides (LPS) are major components of the outer wall of Gram-negative bacteria, are activated either directly or by host-derived mediators such as chemokines, cytokines, complements, and serine proteases, and have an important role in mediating inflammation [3, 4]. Toll-like receptor 4 (TLR4) is a membrane penetrating protein and is a member of the TLR family belonging to the pattern recognition receptor (PRR) family. It is well known that TLR4 primarily recognizes LPS and induces inflammatory and immune responses through the production of inflammatory molecules via the activation of the nuclear factor-*κ*B (NF-*κ*B) and mitogen-activated protein kinase (MAPK) pathways [5–7].

Monocytes have been known to release proinflammatory mediators and regulatory proteins that respond to inflammation and oxidative stress in response to LPS. Heme oxygenase 1 (HO-1) expression is induced in several cell types by various stress stimuli, including proinflammatory cytokines and LPS [8–10]. Nuclear factor erythroid 2-related factor 2 (Nrf-2), a protective factor against oxidative stress, is involved in HO-1 in the anti-inflammatory process [11, 12]. In addition, the induction of HO-1 from Nrf2 activation led to downregulation of the hypersecretion of inflammatory cytokines [13]. Transcription of HO-1 after activation of Nrf-2 is known to inactivate or neutralize NF-*κ*B signaling [14].

THP-1 cells, human leukemia monocytes, have extensively been used for studying the immune response capacity of monocytes and monocyte-derived macrophages in immune system [15, 16]. THP-1 cells are involved in the inflammatory process and have the ability to produce and secrete pro- and anti-inflammatory cytokines [17]. In addition, it was shown that, when stimulated with LPS, THP-1 cells responded with a transcription pattern similar to that of PBMC-derived macrophages [15, 18].

*Pyrus pyrifolia* Nakai is a perennial plant belonging to the family of Rosaceae. Its fruit is edible and is known by many names such as Asian pears, Korean pears, Japanese pears, or Chinese pears. In traditional East Asia medicine, the fruit of *P. pyrifolia* has been used to cure sputum, cough, hangover, fever, and bowel movements while the peels of its fruit have been used to cure abscess, cough, dysentery, swelling, skin diseases, and indigestion [19]. However, there is no known use for research on the effect of the leaves of *P. pyrifolia* on the inflammation. Thus, this study hypothesized that the biological active fraction from the leaves of *P. pyrifolia* has anti-inflammatory property by directly targeting inflammatory signaling while also targeting molecules indirectly associated with inflammation as antibacterial effect by antiradical activity [20]. In this study, the anti-inflammatory effect of PP was investigated in various experiments and LPS was used to induce inflammation in THP-1 monocytes.

## 2. Materials and Methods

### 2.1. Preparation of PP

The plant extract was provided from the Korea Plant Extract Bank at the Korea Research Institute of Bioscience and Biotechnology (Daejeon, Korea). *Pyrus pyrifolia* Nakai was collected from Jeongeup-si, Jeollabuk-do, Korea, in 2002 (a voucher specimen, KRIB 0001364). The leaves of *P. pyrifolia* (110 g) that were dried in the shade and ground into a powder were added to methanol solvent (1 L, HPLC Grade) and extracted through 30 cycles (ultrasonication at 40 KHz, 1500 W, for 15 min and standing for 120 min per cycle) at room temperature using an ultrasonic extractor (SDN-900H, SD-ULTRASONIC Co., Ltd.). After filtration and drying under reduced pressure, *P. pyrifolia* extract (12.63 g) was obtained. The extract was separated by MPLC instrument (ARMEN SPOT-II, Gilson, Middleton, WI, USA) using a reverse phase column (YMC-Pack ODS-AQ HG, 20 × 250 mm, 10 *μ*m, Kyoto, Japan) eluted with MeOH-H_2_O to yield seven column fractions. Complex peaks were analyzed from each column fraction using UPLC-QTOF-MS. The overall process is described in Figures S1 and S2 and Table S1. Among them, PP (a mixture of column fractions nos. 3–5) was selected to investigate its biological activities.

### 2.2. Cell Culture

Human monocyte THP-1 cells, obtained from the American Type Culture Collection, were maintained in RPMI 1640 medium supplemented with 10% fetal bovine serum (S001-07, Welgene, Korea) and 100 U/mL penicillin-streptomycin (Thermo Fisher Scientific, Grand Island, NY) at 37°C incubator with 5% CO_2_.

### 2.3. Cell Viability

CytoX assay was performed to determine the cell viability (CYT3000, LPS Solution, Korea). The cells were seeded at 2 × 10^5^ cells per well in a 96-well plate and incubated with 10, 20, and 40 *μ*g/mL of the total extract or each column fraction for 24 h at 37°C. In addition, to measure the effect of PP, the cells were incubated with PP (1.25, 2.5, 5, 10, 20, and 40 *μ*g/mL) for 1 h and then maintained for 23 h with or without LPS (0.1 *μ*g/mL). After the addition of the CytoX solution into well, THP-1 cells were maintained for 4 h at 37°C. Then, the formazan product was dissolved in dimethyl sulfoxide and the absorbance of each well was determined at 570 nm. The cell viability was expressed as the percentage of surviving cells over the control cells.

### 2.4. Enzyme-Linked Immunosorbent Assay (ELISA)

The supernatant was harvested after treating the THP-1 monocytes with the total extract, column fractions, or PP in the absence or presence of LPS (0.1 *μ*g/mL) for 6 h. The levels of inflammatory cytokines and chemokines were measured following the manufacturer's instructions (555212, 555220, BD Bioscience; DY297, DY208, R&D Systems). The absorbance was measured at 450 nm using a microplate reader (Tecan, Switzerland).

### 2.5. Reverse Transcription PCR (RT-PCR)

The THP-1 monocytes were treated with PP in the absence or presence of LPS (0.1 *μ*g/mL) for 6 h. Total RNA was isolated using the TRIzol™ reagent (Invitrogen, Carlsbad, CA) and the reverse transcription reaction was performed using a kit producing cDNA (Qiagen, Hilden, Germany). Polymerase chain reactions were conducted with specific primers (Table S1). Total RNA extraction, cDNA synthesis, and the mRNA levels of inflammatory molecules were determined as described previously [6].

### 2.6. Western Blotting

After pretreatment of PP for 1 h, cells were stimulated with LPS for 30 min when measuring the p-ERK, ERK, p-JNK, JNK, p-p38, and p38 levels and for 1 h when measuring the p-p65, p65, p-I*κ*B-*α*, and I*κ*B-*α* levels. Additionally, the THP-1 cells were treated with the indicated concentrations of PP or vehicle for 30 min when measuring the p-Nrf2 and Nrf2 levels and for 20 h when measuring HO-1. The isolation of nuclear and cytoplasmic proteins was performed using extraction kit 78833 (Thermo). An equal amount of protein was denatured and resolved on 10% SDS polyacrylamide gels and transferred to the Hybond PVDF membrane (Amersham Biosciences, Piscataway, NJ). The membranes were incubated with blocking buffer (5% skim milk in Tris-buffered saline containing 0.05% Tween-20) for 1 h at room temperature. The following primary antibodies and dilutions were used: phosphorylated (p)-ERK (4370), p-p38 (9211), p-NF-*κ*B (3033), NF-*κ*B (8242), p-I*κ*B-*α* (2859), I*κ*B-*α* (9242, dilutions, 1 : 1000, all from Cell Signaling Technology), p-JNK (sc-6254), JNK (sc-474), ERK (sc-154), p38 (sc-7149), Nrf2 (sc-722), HO-1 (sc-5061), and PCNA (sc-56, dilutions, 1 : 1,000, all from Santa Cruz Biotechnology). They were incubated overnight at 4°C in 5% skim milk. Then, the membranes were washed with TBST for 10 min. In addition, the membranes were incubated with HRP-conjugated secondary antibodies at room temperature for 1 h. Finally, the levels of each protein were detected using an ECL detection system (Bio-Rad Laboratories) according to the manufacturer's instructions. The images were captured with LAS-4000 mini (Fujifilm Co. Ltd., Tokyo, Japan), and the band intensities were analyzed using the ImageJ software (version 1.50e; NIH, MD).

### 2.7. Statistical Analysis

Data are expressed as the mean ± SD. Statistical significance was determined by analysis of two groups using Student's *t*-test (Microsoft excel 2013). The *p* value <0.05 was considered to indicate a statistically significant difference.

## 3. Results and Discussion

### 3.1. Determination of PP from the Extract of *Pyrus pyrifolia* Leaves

Monocytes are closely related to inflammation by producing cytokines and chemokines as a reaction to external stimuli [21]. THP-1 cells have been widely used to study the function of monocytes [22] and are known to have a more mature monocyte phenotype compared to other monocytes [23]. Additionally, THP-1 cells appear to be very similar to PBMC-derived macrophages when stimulated with LPS [16]. Stimulation of monocytes by LPS increases the expression of TLR4, leading to the generation of inflammatory molecules. These proinflammatory factors have an important role in inflammatory diseases [21], including atherosclerosis [24], rheumatoid arthritis [25], and sepsis [26]. Thus, it is emphasized to find a pharmacological compound that does not have cytotoxicity and undesirable effects and exhibits such anti-inflammatory effects.

**Figure 1:**
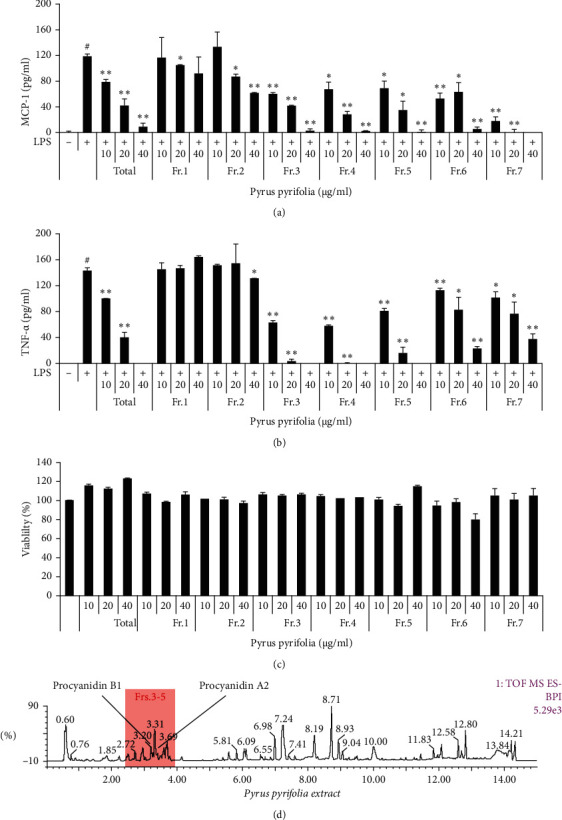
Effects of the total extract and column fractions from the leaves of *Pyrus pyrifolia* on cytokines production and cell viability. THP-1 cells were maintained with the presence or absence of 0–40 *μ*g/mL and with or without LPS. MCP-1 (a) and TNF-*α* (b) were measured by ELISA and the determination of cell viability (c) was performed using the CytoX kit. The complex peaks of *Pyrus pyrifolia* were assessed with UPLC-QTOF-MS (d). Data are expressed as the mean ± SD. ^#^*P* < 0.05 versus negative control group; ^*∗*^*P* < 0.05 and ^*∗∗*^*P* < 0.01 versus LPS only group.

Therefore, we performed the CytoX assay and cytokine assays to evaluate the anti-inflammatory properties of the crude extract and column fractions of *P. pyrifolia*. The total extract and the column fractions were nontoxic at 40 *μ*g/mL except for fraction no. 6. They dose-dependently decreased the release of MCP-1 and TNF-*α* in the LPS-stimulated THP-1 cells except for fraction no. 1. In particular, fractions nos. 3, 4, and 5 have significantly decreased the release of LPS-induced TNF-*α* and MCP-1 in THP-1 cells (Figures 1(a)–1(c)). Thus, fractions nos. 3–5 (PP) were used in the rest of the experiments in this study unless otherwise noted (Figure 1(d)). As shown in Supplementary Figure 2, by analyzing the peaks identified in the UPLC chromatogram of PP, it was mainly identified as a mixture of 20 compounds that included caffeoylquinic acid, dicaffeoylquinic acid, procyanidin B1, C1, A2, and catechin (Supplementary Table 1). MCP-1 and TNF-*α* assays were done to determine whether the cytokine inhibitory activity of the PP was caused by procyanidin A2 and B1. However, they did not show a stronger cytokine inhibitory activity than that of the PP (data not shown).

### 3.2. Inhibitory Effect of PP on Secretion of Proinflammatory Molecules in LPS-Stimulated THP-1 Cells

It has been known that the increased MCP-1 is associated with the infiltration of monocytes and macrophage to inflammatory site, and TNF-*α* induces the production of other cytokines and chemokines. ELISA was performed for MCP-1 and TNF-a as well as IL-8 and IL-6 to examine the inhibitory effect of PP on the production of cytokines and chemokine.

**Figure 2:**
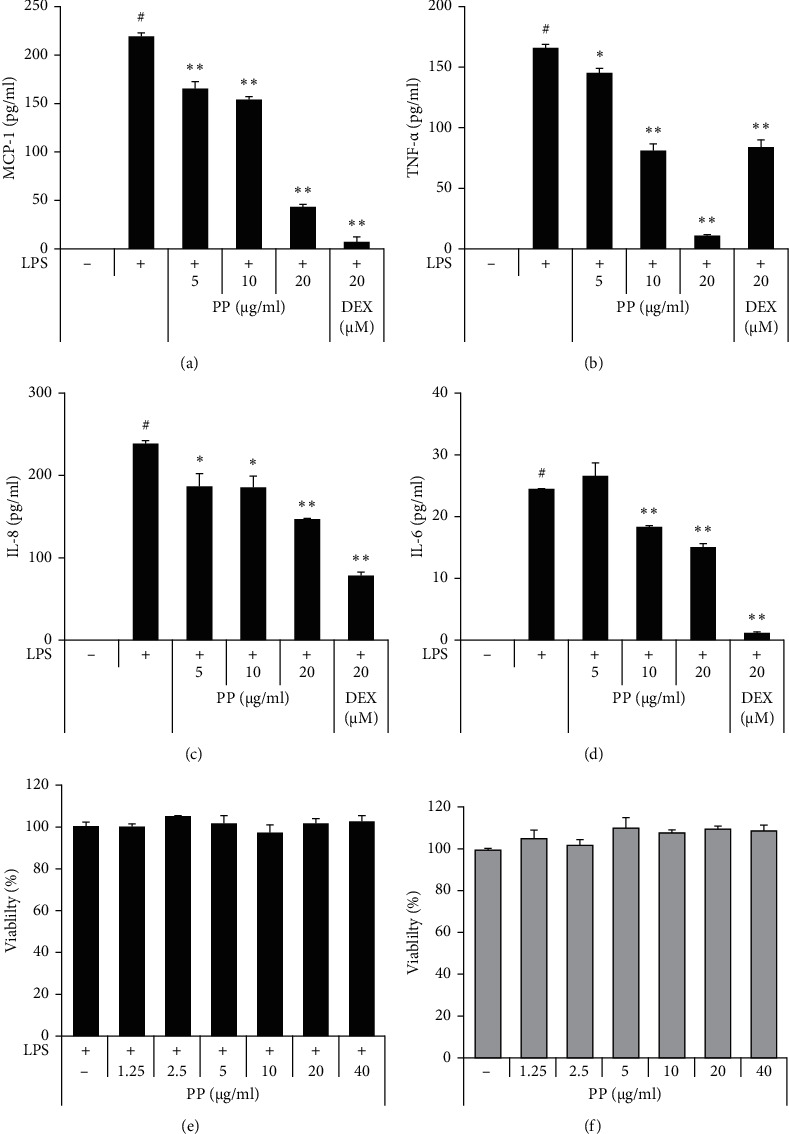
Effects of PP on inhibition of proinflammatory molecules in LPS-stimulated THP-1 cells. THP-1 cells were pretreated with PP (5–20 *μ*g/mL) or DEX (20 *μ*M) for 1 h and maintained with 0.1 *μ*g/mL LPS for 6 h. The expressions of MCP-1 (a), TNF-*α* (b), IL-8 (c), and IL-6 (d) were measured by ELISA. After treatment for 24 h with (e) or without LPS (f), cell viability was determined. Data are expressed as the mean ± SD. ^#^*P* < 0.05 versus negative control group; ^*∗*^*P* < 0.05 and ^*∗∗*^*P* < 0.01 versus LPS only group.

As shown in Figure 2, the production levels of cytokines and chemokines, such as TNF-*α*, MCP-1, IL-8, and IL-6 were upregulated in cells treated with LPS only shown in Figure 1. On the other hand, in cells cotreated with PP and LPS, this increase of cytokines and chemokine significantly reduced in a dose-dependent manner (Figures 2(a)–2(d)).

Cell viability was measured with 0–40 *μ*g/mL PP, which was a wider range than the concentrations used to measure the cytokine inhibitory effect. PP had no cytotoxic effects at any of the concentrations used with or without LPS (Figures 2(e) and 2(f)).

### 3.3. Inhibitory Effect of PP on mRNA Expression of Proinflammatory Molecules in LPS-Stimulated THP-1 Cells

**Figure 3:**
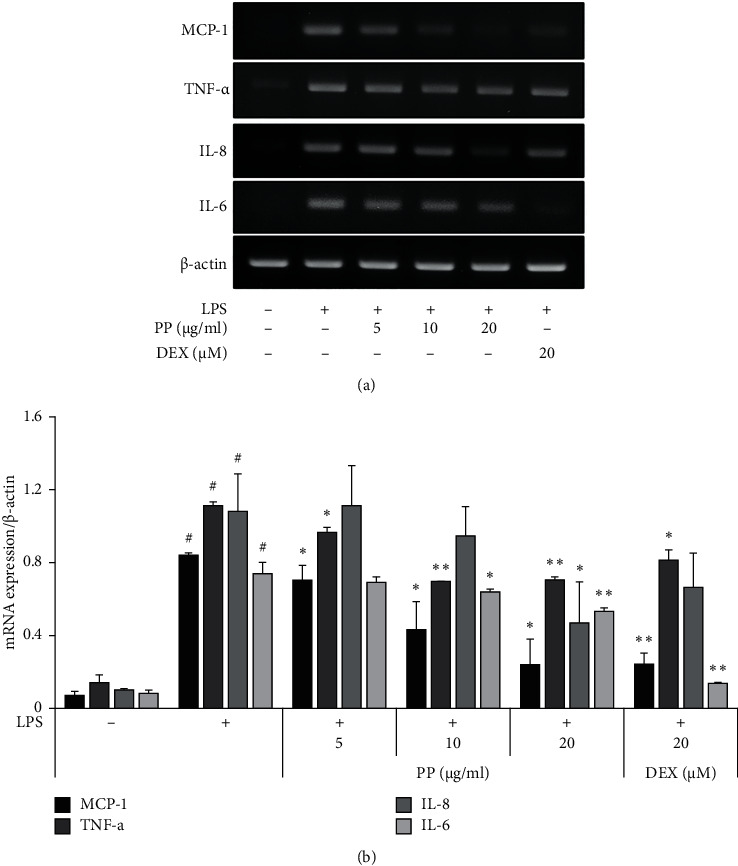
Effects of PP on mRNA expression of proinflammatory molecules in LPS-stimulated THP-1 cells. THP-1 cells were pretreated with PP (5–20 *μ*g/mL) or DEX (20 *μ*M) for 1 h; then cells were maintained with 0.1 *μ*g/mL LPS for 6 h. The total RNA was isolated, and the mRNA levels encoding TNF-*α*, MCP-1, IL-8, and IL-6 were determined by RT-PCR (a). Relative mRNA expression was calculated using *β*-actin as a general housekeeping gene (b). Data are expressed as the mean ± SD. ^#^*P* < 0.05 versus negative control group; ^*∗*^*P* < 0.05 and ^*∗∗*^*P* < 0.01 versus LPS only group.

The mRNA levels of proinflammatory molecules were evaluated using RT-PCR to confirm whether the change in cytokine release is due to RNA expression. The gene expression levels of TNF-*α*, MCP-1, IL-8, and IL-6 were increased in cells treated with LPS alone; however, these expression levels were significantly reduced in a dose-dependent manner by the treatment with PP. In particular, it was confirmed that the expression of MCP-1 was most noticeably reduced (Figure 3(b)). The results showed that the inhibition in the mRNA expression of each molecules was similar to those observed for their protein expression levels.

### 3.4. Inhibitory Effect of PP on LPS-Induced MAPK Activation in THP-1 Cells

**Figure 4:**
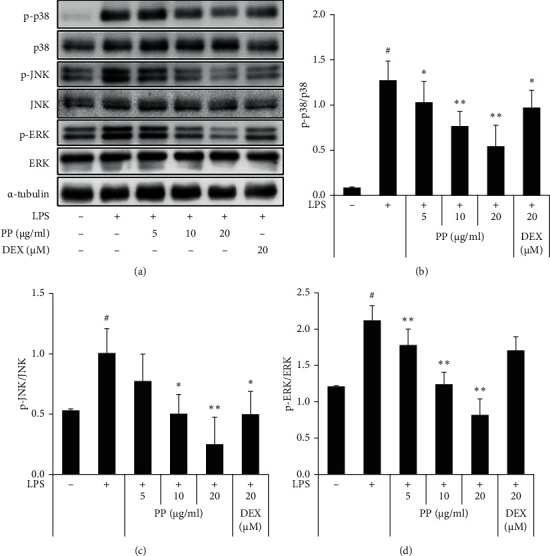
Effect of PP on the activation of MAPK in THP-1 cells. THP-1 cells were treated with PP (5–20 *μ*g/mL) or DEX (20 *μ*M) for 1 h and maintained with 0.1 *μ*g/mL LPS for 30 min. The protein levels of total or phosphorylated (p) ERK, JNK, and 38 were measured by western blotting (a) and quantified (b–d). Data are expressed as the mean ± SD. ^#^*P* < 0.05 versus negative control group; ^*∗*^*P* < 0.05 and ^*∗∗*^*P* < 0.01 versus LPS only group.

The MAPK pathway consists of a group of signaling molecules that have an important role in the inflammatory process and have the ability to direct cellular stimulation to external stimuli. MAPK signaling refers to the activation of p38, JNK, and ERK [27–30]. In particular, MAPKs, including ERK, JNK, and p38, are known to be activated by LPS [31]. Therefore, the phosphorylation of the MAPKs was detected by western blot analysis to evaluate the effect of PP on MAPK signaling pathway.  Figure 4 shows that LPS stimulation of THP-1 cells led to upregulation of ERK, JNK, and p38 phosphorylation, and PP effectively downregulated their phosphorylation (Figure 4). These results suggest that the inhibitory effect of PP on proinflammatory molecules may have been associated with the downregulation of the MAPK pathways.

### 3.5. Inhibitory Effects of PP on LPS-Induced NF-*κ*B Activation in THP-1 Cells

TLR4 signaling is essential for the immune response, which leads to the activation of inflammatory transcription factors such as NF-*κ*B [32]. NF-*κ*B, which consists of the p65 and p50 subunits, leads to expression of inflammatory genes [33]. These subunits block I*κ*B-*α* in the normal state; however, when an inflammatory reaction occurs, I*κ*B-*α* is phosphorylated and degrades during NF-*κ*B activation [34]. LPS stimulates the expression of proinflammatory cytokines and chemokines by NF-*κ*B activation [35].

**Figure 5:**
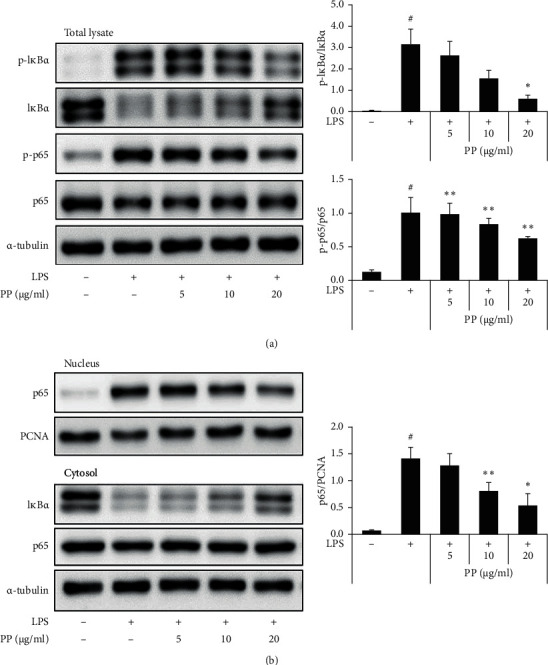
Effects of PP on LPS-induced NF-*κ*B transcriptional activity in THP-1 cells. THP-1 cells were pretreated with PP (5–20 *μ*g/mL) for 1 h; then cells were maintained with 0.1 *μ*g/mL LPS for 1 h. The levels of I*κ*B-*α* and p65 phosphorylation and I*κ*B-*α* degradation were detected with whole-cell lysates by western blot analysis (a). The levels of NF-*κ*B translocation were detected by western blot separated into the cytosol and nucleus (b). Data are expressed as the mean ± SD. ^#^*P* < 0.05 versus negative control group; ^*∗*^*P* < 0.05 and ^*∗∗*^*P* < 0.01 versus LPS only group.

THP-1 cells were pretreated with PP or DEX for 1 h before being treated with LPS to explain the molecular mechanism of anti-inflammatory action of PP. The levels of I*κ*B-*α* degradation and I*κ*B-*α* and p65 phosphorylation were suppressed by PP in a concentration-dependent manner (Figure 5(a)). Moreover, the LPS-induced NF-*κ*B nuclear translocation was inhibited by PP in the THP-1 cells (Figure 5(b)).

These results indicate that the decreased phosphorylation of MAPK by PP may have an anti-inflammatory effect, which could be closely associated with inhibition of NF-*κ*B translocation. Therefore, this study confirmed that PP not only suppressed inflammatory molecules in the LPS-stimulated THP-1 cells but also reduced I*κ*B-*α* degradation by inhibiting the phosphorylation of I*κ*B-*α* and p65. Moreover, this study indicated that PP has anti-inflammatory effects by inhibiting NF-*κ*B signaling (Figure 5). Moreover, this study also showed that PP suppresses inflammatory molecules by attenuating MAPK and NF-*κ*B signaling in the LPS-stimulated THP-1 cells.

### 3.6. Effect of PP on HO-1 Induction in THP-1 Cells

**Figure 6:**
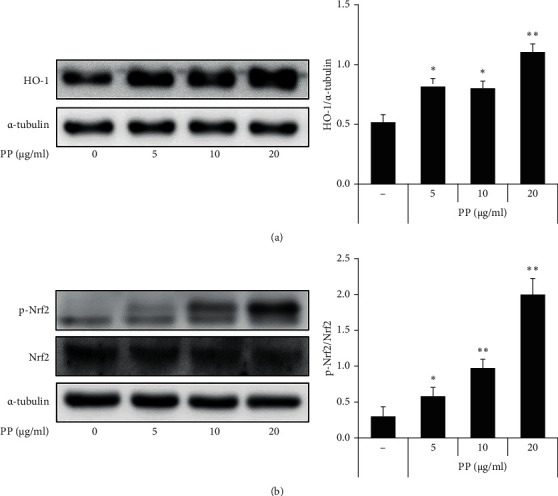
Effect of PP on HO-1 induction in THP-1 cells. HO-1 was detected after treatment with PP (5–20 *μ*g/mL) in the THP-1 cells for 20 h (a). Nrf-2 was detected after treatment with PP (5–20 *μ*g/mL) in the THP-1 cells for 30 min (b). The expression levels of each protein were determined using western blot and data are expressed as the mean ± SD. ^*∗*^*P* < 0.05 and ^*∗∗*^*P* < 0.01 versus control group.

It has been reported that HO-1 is expressed by Nrf-2 activation [36]. HO-1 induction affects the reduction of inflammatory response by controlling NF-*κ*B activation and the generation of inflammatory molecules [37, 38]. Thus, HO-1 was measured after treatment with PP in THP-1 cells for 20 h to evaluate the antioxidant activity. Moreover, p-Nrf2 and Nrf2 were detected after treatment with PP in the THP-1 cells for 30 min. The HO-1 and Nrf-2 expression levels were significantly increased by PP. These results show that PP increased the HO-1 expression by activating Nrf-2 in the THP-1 cells (Figure 6).

## 4. Conclusions

**Figure 7:**
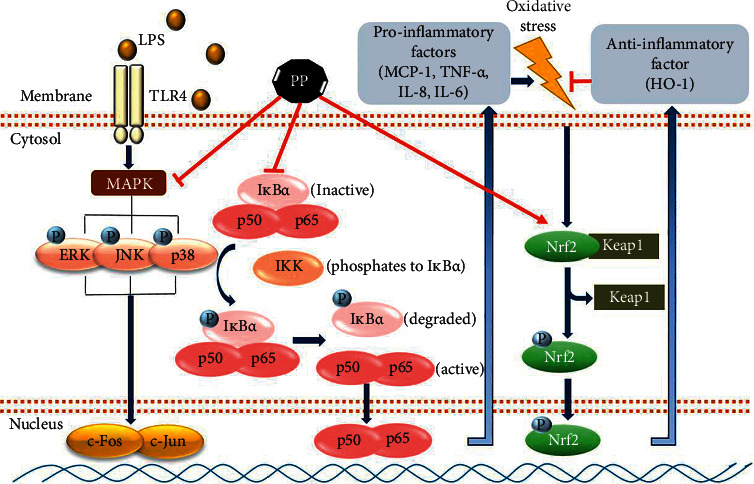
Scheme of the anti-inflammatory effects of PP, the active fraction from the leaves of *Pyrus pyrifolia*, by regulating the MAPK and NF-*κ*B pathway in LPS-stimulated THP-1 cells.

In summary, PP exerted suppressive effect on inflammatory molecules by inhibiting the MAPK and NF-*κ*B signaling pathway. Moreover, PP inhibited oxidative stress through the activation of Nrf-2. Taken together, these findings show the possibility that PP could be used as a potential therapeutic treatment against inflammation (Figure 7). Moreover, this study shows the need for further studies on isolated compounds from PP to understand their anti-inflammatory activity.

## Data Availability

All data supporting the findings are included within the article and supplementary materials.

## Supplementary Materials

Supplementary Materials Figure S1: MPLC chromatogram of the extract of *Pyrus pyrifolia* leaves. Figure S2: UPLC-QTOF-MS chromatogram of the extract of the *Pyrus pyrifolia* leaves. Table S1: tentative identification of the major chromatogram peaks for the *Pyrus pyrifolia* leaves. Table S2: primers for PCR.
