# Supplementary material for: Anti-Inflammatory Effects of the Fraction from the Leaves of Pyrus pyrifolia on LPS-Stimulated THP-1 Cells
Source: Evid Based Complement Alternat Med. 2021 Aug 25;2021:4946241. doi: 10.1155/2021/4946241 (PMC8413047; doi:10.1155/2021/4946241)
Supplement: Supplementary Materials — Figure S1: MPLC chromatogram of the extract of Pyrus pyrifolia leaves. Figure S2: UPLC-QTOF-MS chromatogram of the extract of the Pyrus pyrifolia leaves. Table S1: tentative identification of the major chromatogram peaks for the Pyrus pyrifolia leaves. Table S2: primers for PCR. [file 4946241.f1.docx]

**Supplementary Materials**

**Anti-inflammatory effects of the fraction from the leaves of**

***Pyrus pyrifolia* on LPS-stimulated THP-1 cells**

Gilhye Lee,^1,2^ Jung-Hee Kim,^1^ Hyun-Jae Jang^1^, Ji-Won Park,^1^ Jae-Won Lee,^1^ Ok-Kyoung Kwon^1*^, Jae-Hong Kim,^2,*^ and Kyung-Seop Ahn^1,*^

^1^ Natural Medicine Research Center, Korea Research Institute of Bioscience and Biotechnology, Cheongju-si 28116, Republic of Korea
^2^ College of Life Science and Biotechnology, Korea University, Seoul, 02841, Republic of Korea

^*^Correspondence should be addressed to Ok-Kyoung Kwon; dooli@kribb.re.kr, Jae-Hong Kim; jhongkim@korea.ac.kr, and Kyung-Seop Ahn; ksahn@kribb.re.kr

***1. Preparation of PP***

The plant extract used in this study was obtained from the Korea Plant Extract Bank at the Korea Research Institute of Bioscience and Biotechnology (Daejeon, Korea). The leaves of *Pyrus pyrifolia* Nakai were collected from Jeongeup-si, Jeollabuk-do, Korea in 2002. A voucher specimen (KRIB 0001364) is kept in the herbarium of the Korea Research Institute of Bioscience and Biotechnology. The leaves of *P. pyrifolia* (110 g) that were dried in the shade and ground into a powder were added to methanol solvent (1 L, HPLC Grade) and extracted through 30 cycles (40 KHz, 1500 W, 15 min. ultrasonication per 120 min. standing per cycle) at room temperature using an ultrasonic extractor (SDN-900H, SD-ULTRASONIC CO., LTD). After filtration and drying under reduced pressure, *P. pyrifolia* extract (12.63 g) was obtained. The extract was separated by MPLC instrument (ARMEN SPOT-II, Gilson, Middleton, WI, USA) using a reverse phase column (YMC-Pack ODS-AQ HG, 20 × 250 mm, 10 μm, Kyoto, Japan) eluted with MeOH–H_2_O to yield seven column fractions. Complex peaks were analyzed from each column fractions using UPLC-QTOF-MS. The overall process is described in Figures S1 and S2, and compounds were identified (Table S1).


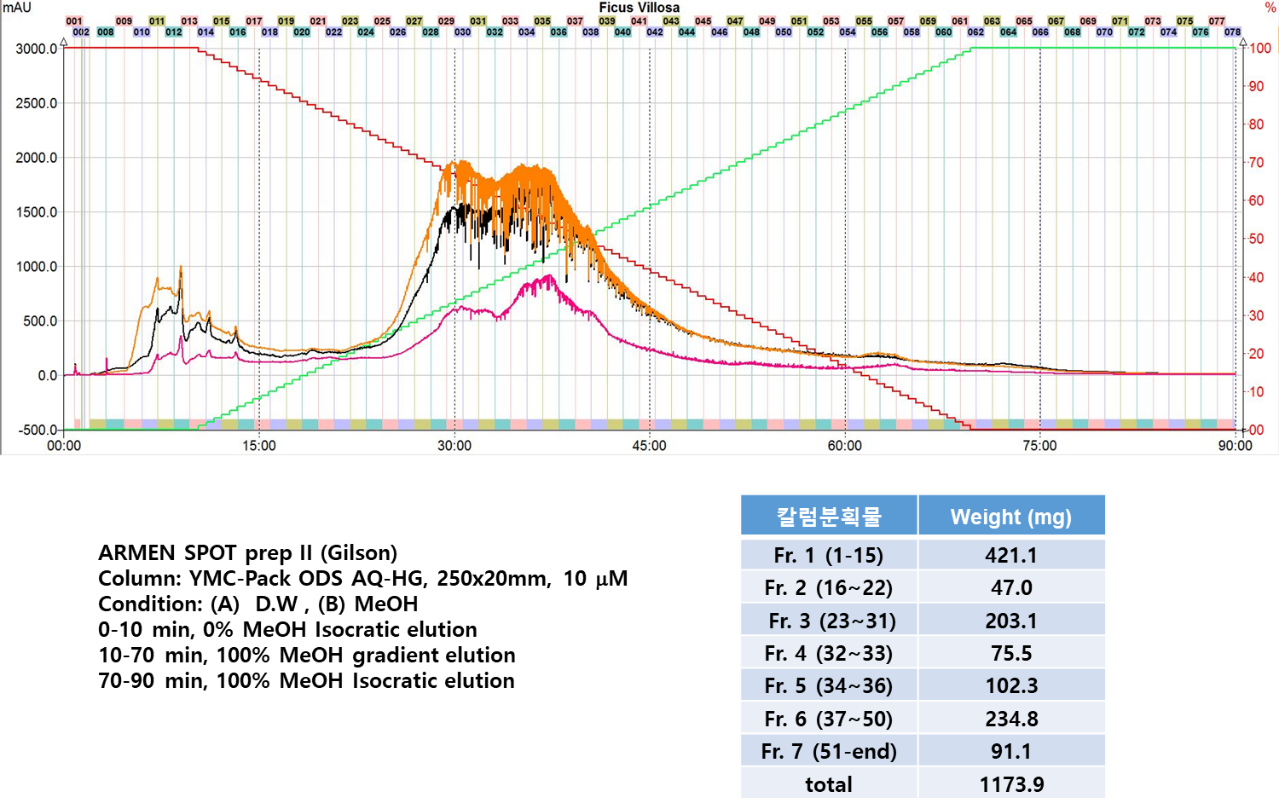
Figure S1. MPLC chromatogram of *Pyrus pyrifolia* leaves extracts


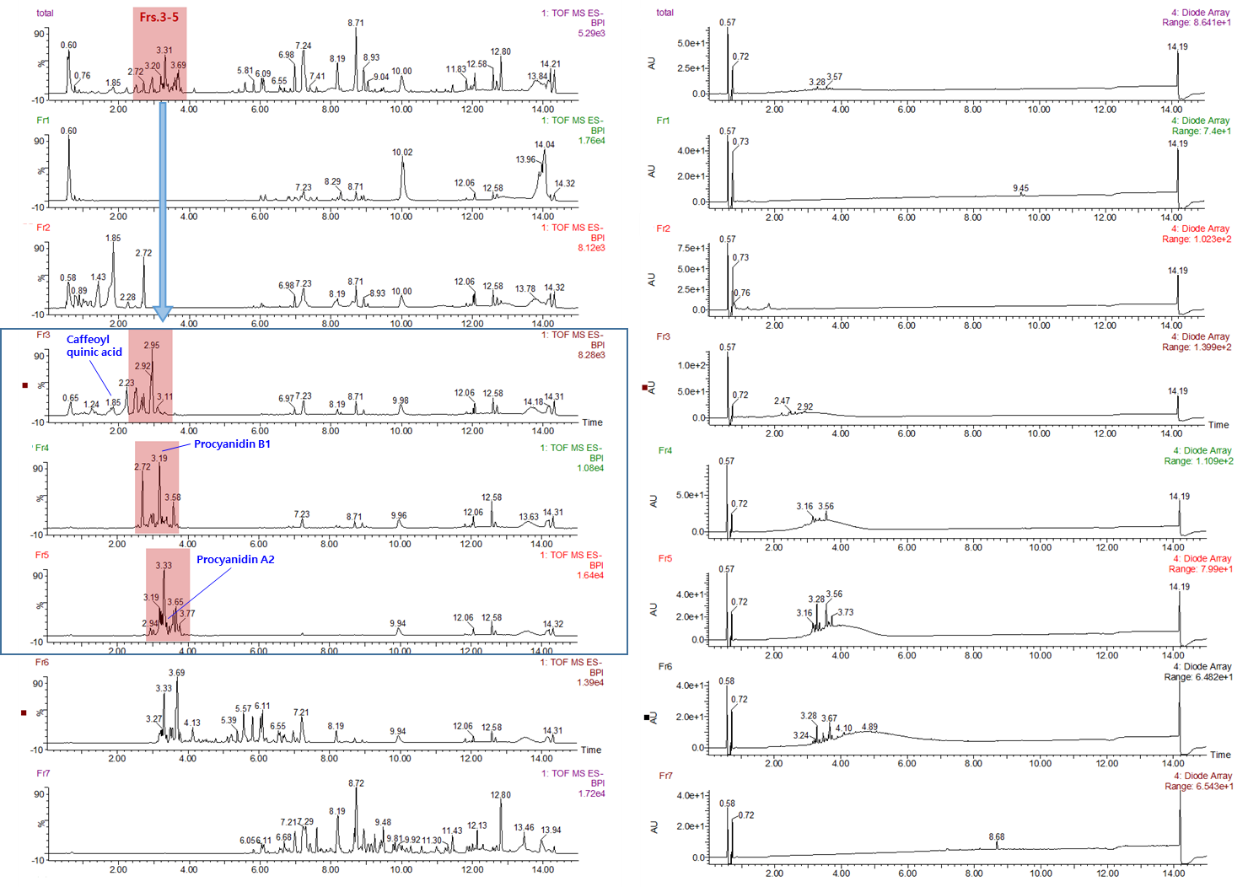


Figure S2. UPLC-QTOF-MS chromatogram of the extract of the *Pyrus pyrifolia* leaves.

Complex peaks were analyzed from each column fractions using UPLC-QTOF-MS. The extracts were separated by MPLC (ARMEN SPOT-II, Gilson, Middleton, WI, USA) using reverse phase column (YMC-Pack ODS-AQ HG, 20 × 250 mm, 10 μm, Kyoto, Japan) eluted with MeOH–H_2_O to yield seven column fractions. Some peaks of each column fractions were tentatively identified by using UPLC-QTOF-MS.

Table S1. Tentatively identification of the major chromatogram peaks for the *Pyrus pyrifolia* leaves


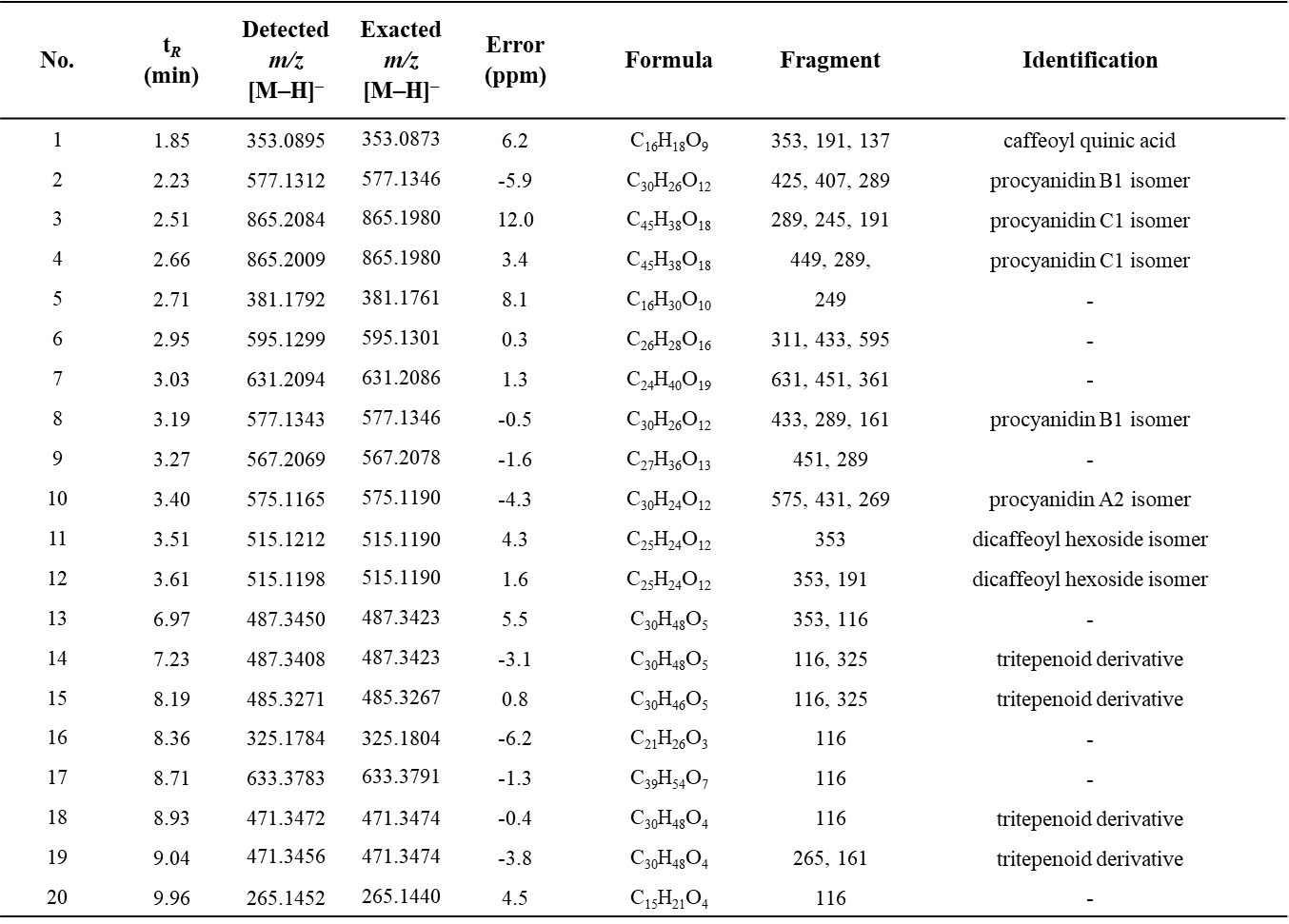


***2. Reverse transcription-polymerase chain reaction (RT-PCR).***

The THP-1 monocytes were treated with PP in the absence or presence of LPS (0.1 μg/mL) for 6 h. Total RNA was isolated using the TRIzol™ reagent (Invitrogen Life Technologies, Carlsbad, CA, USA) and the reverse transcription reaction was performed using a kit producing cDNA (Qiagen, Hilden, Germany). Polymerase chain reactions were conducted with specific forward and reverse primers (Table S2). The GoTaq® G2 Green Master Mix (cat. no. M7823; Promega, Madison, USA) was used. PCR products were separated on a 1.5% agarose gel with the RedSafe™ kits (Intron Biotechnology, Inc., Gyeonggi-do, Korea). Images of the gels were captured with an Olympus C4000 zoom camera system (Olympus, Tokyo, Japan) and were analyzed by the Image J software (version 1.50e; National Institutes of Health, Bethesda, MD, USA).

Table S2. Primers for PCR

| **Name** |  | **Sequence** | **Size** |
| --- | --- | --- | --- |
| **MCP-1** | **forward** | **5’-** **TGCAGAGGCTCGCGAGCTA-3’** | **137bp** |
|  | **reverse** | **5’-** **CAGGTGGTCCATGGAATCCTGA-3’** |  |
| **TNF-α** | **forward** | **5’-** **TCAACCTCCTCTCTGCCATC -3’** | **317bp** |
|  | **reverse** | **5’-** **CCTAAGCCCCCAATTCTCTT -3’** |  |
| **IL-8** | **forward** | **5’-** **ATGACTTCCAAGCTGGCCGTGGCT -3’** | **299bp** |
|  | **reverse** | **5’-** **TTATGAATTCTCAGCCCTCTTCAAAAA -3’** |  |
| **IL-6** | **forward** | **5’-** **GAC AGC CAC TCA CCT CTT CA -3’** | **124bp** |
|  | **reverse** | **5’-** **AGTGCCTCTTTGCTGCTTTC -3’** |  |
| **β –actin** | **forward** | **5’-** **CAT GTA CGT TGC TAT CCA GGC -3’** | **250bp** |
|  | **reverse** | **5’-** **CTC CTT AAT GTC ACG CAC GAT -3’** |  |
